# Inflammation to Infertility: Panoramic View on Endometriosis

**DOI:** 10.7759/cureus.11516

**Published:** 2020-11-16

**Authors:** Huda A Mohammed Rasheed, Pousette Hamid

**Affiliations:** 1 Research, California Institute of Behavioral Neurosciences & Psychology, Fairfield, USA; 2 Neurology, California Institute of Behavioral Neurosciences & Psychology, Fairfield, USA

**Keywords:** endometriosis, infertility, inflammation and infertility

## Abstract

Endometriosis is a disease caused by the implantation of endometrial glands and stroma outside the uterine cavity. It affects 10% of the reproductive-age women this means that 190 million women are affected worldwide. The definitive diagnosis requires surgical exploration or a laparoscopy which is of a high expense. The pathogenesis of the disease is heterogeneous and poorly understood despite the progress in the research field. Infertility is one of the main symptoms of Endometriosis. The mechanism behind this remains unclear. Literature suggests that Endometriosis reduces implantation capacity, increases the risk of pregnancy loss, and causes anatomical obstruction imposed by endometriotic lesions. The disease has a high burden to it by decreasing the quality of women's life and imposing negative consequences for their productivity, social life, and emotional wellbeing. Since inflammation is considered the hallmark of endometriosis, it is worth looking at the mechanism of how inflammation is linked to infertility in endometriosis patients. In this study, we summarized the recent finding of how inflammation can affect oocyte, endometrium, hormones, and sperm.

## Introduction and background

Endometriosis affects 190 million women worldwide, around 10% of the reproductive-age women. Endometriosis is a disease caused by endometrial glands and stroma outside the uterine cavity, mostly in the ovaries and pelvic peritoneum. Other sites including, abdominal wall, Fallopian tube (FT), bowels, bladder, cervix, and vagina [1]. Patients present with chronic pelvic pain, dyspareunia, cyclic menstrual pain, dyschezia, and infertility [2].

The definitive diagnosis requires surgical exploration or a laparoscopy. The diagnosis is made based on the clinical history and physical examination in which most patients show tenderness on palpation of the posterior fornix. Other physical findings are uterine and adnexal tenderness, nodular uterosacral ligament, and pelvic mass. Other diseases that can cause pelvic pain should be ruled out, like adenomyosis, pelvic adhesion, gastrointestinal or urological disease [3]. The high expense of the definitive diagnosis, the non-specific symptoms of endometriosis, and insufficient definitive biomarkers render the disease's diagnosis delayed. Treatments include: hormonal therapy and nonsteroidal for symptomatic endometriosis [3], and surgical therapy is used for infertile women [2]. However, because these treatments do not target the disease's mechanism, they are not curative [3,4] and have limited efficacy as endometriosis often recurs [3]. 

The mechanism of endometriosis

This disease's pathogenesis is explained by multiple theories, both old and new, in the research field, suggesting it is a multifactorial and heterogeneous disease. Lagna et al. [4] revised the postulated theories through the literature and divided them into two categories :

A. The Transplantation Theory

This category's concept is that endometriosis tissue exists from the local tissue by metaplasia or by embryological origin [5]. It contains the celomic metaplasia in which the peritoneal mesothelial cell transforms into glandular endometrium. This theory has been postulated in women with müllerian duct defects [6]. Moreover, the mullerianosis theory in which endometriosis like tissue originate from primitive endometrial tissue that was misplaced along the migration pathway of the mullerian duct [7].

B. *The In-situ Theory*

It is based on the concept that endometriosis originates from eutopic endometrium and metastasize to other sites by the hematogenous, lymphatic, or iatrogenic spread. This category includes the implantation theory, also called Sampson, who proposed a retrograde flow of the menstrual blood and endometrial tissue through the fallopian tubes into the peritoneal cavity is the first step in the development of the disease [8]. Brosens and Benagiano added to this by suggesting that the whole process starts when girls are born. They develop neonatal hormonal deprivation bleeding, which can happen in a retrograde fashion. These deposits would remain inactive until puberty [9].

A genetic basis for endometriosis development is suggested by the reports of familial aggregation and endometriosis's high risk in those with an affected first-degree relative [10]. Furthermore, a new stem cell theory is proposed in the literature; stem cells, which can differentiate to several tissue types and replenish themselves as well, have been recently identified to be involved in the development of ectopic endometrial lesions [11].

Despite the progress in the research field, the mechanisms of endometriosis remain poorly understood. In the current literature, there is growing evidence that all these factors lead to the activation of a local inflammatory microenvironment, which supports endometriosis to grow and provide mediators responsible for its two prominent pain symptoms and infertility [12].

Infertility is one of the common complications of endometriosis [2]. Infertility is a disease of the reproductive system defined by the failure to achieve pregnancy after 12 months or more of regular unprotected sexual intercourse. Infertility and endometriosis have a complicated relationship as asymptomatic endometriosis is usually detected during an infertility evaluation, and among infertile women, endometriosis is the most observed gynecologic disease. In an updated study, the risk of developing infertility in endometriosis settings was shown to be only in those who are <35 years of age [13]. 

The mechanism behind this remains unclear. Literature suggests that endometriosis reduces implantation capacity, increases the risk of pregnancy loss, and causes anatomical obstruction imposed by endometriotic lesions [14]. Additionally, the disease decreases the quality of women's life by imposing negative consequences for their productivity, social life, and emotional wellbeing [15]. It has been further classified according to its effects, from ovarian dysfunction and tubal defects to uterine or peritoneal causes [16]. Regarding infertility treatment, medical therapy has not been proven to be beneficially effective [17]. The hormones suppress ovarian function and create a contraceptive state and atrophy to the endometrial lining rather than a fertile state [18]. Surgical treatment of endometriosis involving resection of endometriotic tissue has a risk of ovarian damage that will further worsen the case. [17]. 

**Figure 1:**
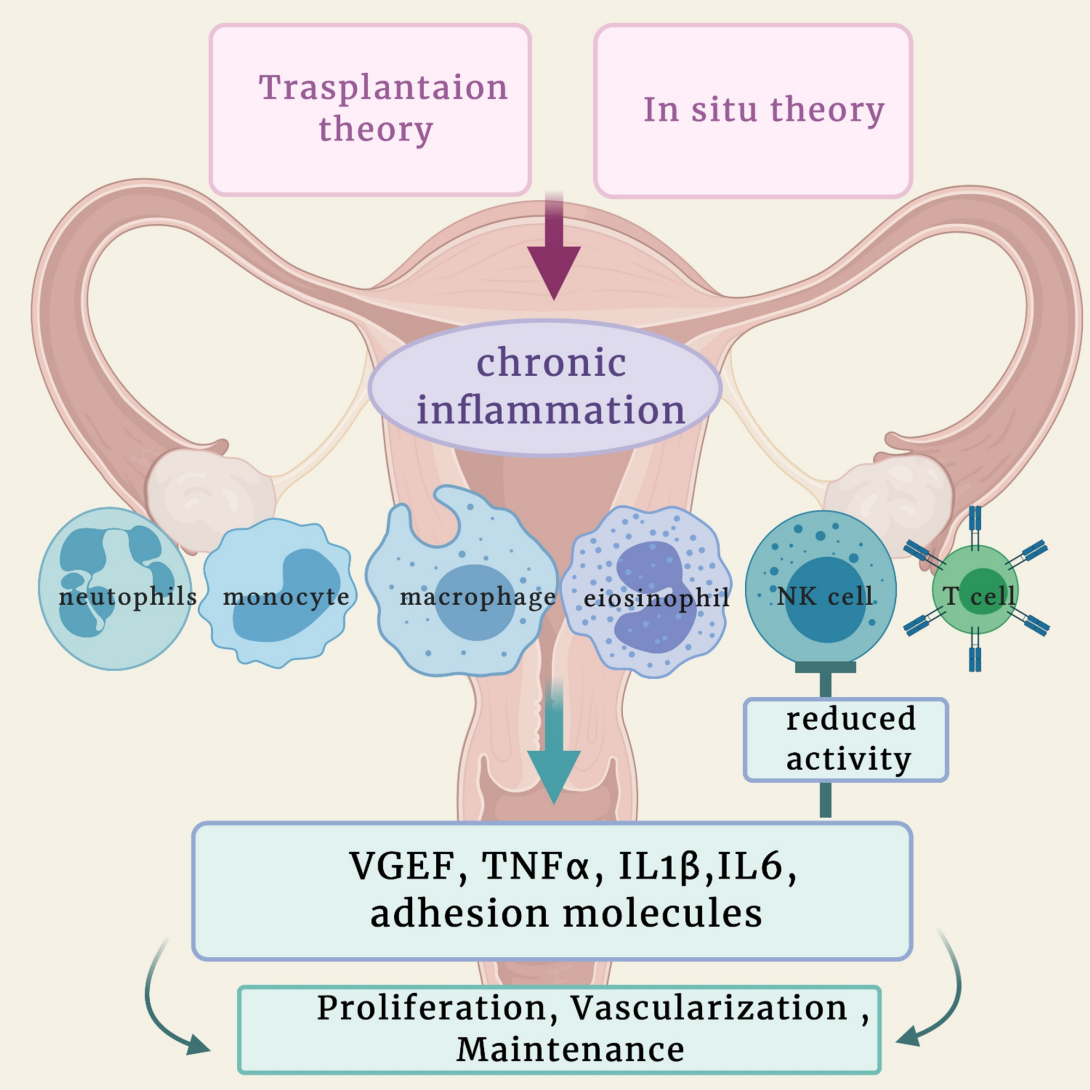
Inflammatory mechanism in endometriosis. NK cell: natural killing cell; VEGF: vascular endothelial growth factor; TNFᵅ: tumor necrosis factor; IL1β: interleukin one beta; IL6: interleukin six Created with BioRender.com and adapted from [23]

Inflammation has a vital role in endometriosis's pathophysiology, responsible for the local and systemic symptoms and signs. Thus inflammatory mediators may serve as biomarkers for diagnosis or the target of treatment [19]. The endometrial tissue produces a localized inflammatory response and inflammatory mediators (cytokines, chemokines, and prostaglandin). The chemokines will attract neutrophils, macrophages, monocytes, eosinophils, and T cells [20]. Neutrophil in the abdominal cavity can secrete vascular endothelial growth factor (VEGF), a significant pro-angiogenic factor, increasing its level in the peritoneal fluid in endometriosis. Therefore, neutrophils may support the growth of endometriotic lesions by secreting VEGF [21]. The local natural killer cell activity is impaired, which is probably why the endometriotic cell escaped the immune system [22]. There is a decreased phagocytic activity in the macrophage and an increase in the inflammatory mediators. Tumor necrosis factor-alpha (TNF-α), interleukin one beta (IL-1β), and interleukin six (IL-6) are all elevated as well as the angiogenic factors (VGEF), the growth factors, and adhesion molecules [23,24] all of which play a positive role in the occurrence, maintenance, and progression of endometriosis. An increase in the number of white blood cells in the peritoneal fluid of women with endometriosis was observed. The finding above supports the inflammatory hypothesis in endometriosis (Figure 1) [25].

Recent studies are searching for a non-invasive diagnostic biomarker, and since inflammation is the hallmark of the disease, inflammatory markers can be of use. They found that IL-6, IL-10, IL-13, and TNF-α are highly expressed in the peritoneal fluid of endometriosis patients. Thus, these inflammatory factors (IL-6, IL-10, IL-13, and TNF-α) can be used as essential reference indexes for endometriosis diagnosis complicated with infertility [26]. Ca 125 is the primary biomarker used now; however, it is only used for follow-up rather than diagnosis. It is not sensitive nor specific enough to be used in screening. A recent study exploring this biomarker's diagnostic value with infertility and finding other valuable biomarkers hypothesized that the combined use of IL6 and IL8 with CA125 has a higher predictability value than CA125 alone [27].

These potential biomarkers will serve as an excellent tool for early diagnosis, thus reducing the cost of the surgical intervention, detect the disease early, allowing for better management, fewer complications, and improving the outcomes for pain and infertility. With endometriosis having a high cost of illness burden in Europe, the UK, and the USA and the majority of costs coming from reductions in productivity [28], this highlights the need for more research in this area of medicine [3].

This review aims to provide more insight into how inflammation can cause infertility in endometriosis patients.

## Review

Methods

Search Strategy 

A literature search in PMC and PubMed was carried out using the following keywords: "endometriosis and infertility," "inflammatory mechanism of endometriosis-induced infertility," "inflammation of endometriosis and infertility." Study selection was based on year of publication (last five years), language (English only), model (humans only), open accesses, and all types of studies were included as long as they were relevant to the study. We expanded our search to older articles through referencing for a more detailed understanding, and some closed access articles were obtained.

Results

We screened 80 articles by abstract and excluded 35 as they were irrelevant and would not contribute to our study. We included five articles through references. Most of our studies were reviews that addressed endometriosis infertility with different mechanisms and associations. In Table 1, the reviews conclude that inflammation has a negative impact on the fertility of endometriosis patients at different molecular levels.

**Table 1:** Study characteristic table

author	Journal	Year	Population	conclusion	Type of the study
Miller et al. [29]	Oncotarget	2016	Infertile Endometriosis patient	Negative effect on oocyte, sperm, embryo quality and receptivity	Narrative review
Vannuccini et al. [30]	Human Reproduction Update	2016	Infertile Women with reproductive disorders (Endometriosis, adenomyosis, polycystic ovarian syndrome)	Negative effect on receptivity (implantation)	Narrative review
Sanchez et al. [31]	Journal of Ovarian Research	2017	Infertile Endometriosis patients	Negative impact on oocyte quality	Narrative review
Lessey and Kim [14]	Fertility and sterility	2017	Endometriosis patients	Negative impact on receptivity	Narrative review
Máté, Bernstein, and Török. [32]	Frontiers in Endocrinology	2018	Endometriosis patient with In vitro fertilization	Negative impact on oocyte, sperm, embryo	Narrative review
Lin et al. [18]	International Journal of Molecular Sciences	2018	Infertile Endometriosis patients	Negative effect on hormones, ovaries, receptivity, and implantation	Narrative review
Yu et al. [33]	Molecular Human Reproduction	2019	Case: laparoscopic confirmed endometriosis Control: fertile women	Negative impact on receptivity (decidualization)	Case-control
García-Gómez et al. [34]	Frontiers in Endocrinology	2020	Endometriosis patients	Negative impact on hormonal signaling pathways	Narrative review
Hill et al. [35]	Journal of Clinical Medicine	2020	Endometriosis patients	Negative effect on sperm, oocyte, and embryo ( tubal function)	Narrative review

Discussion

**Figure 2:**
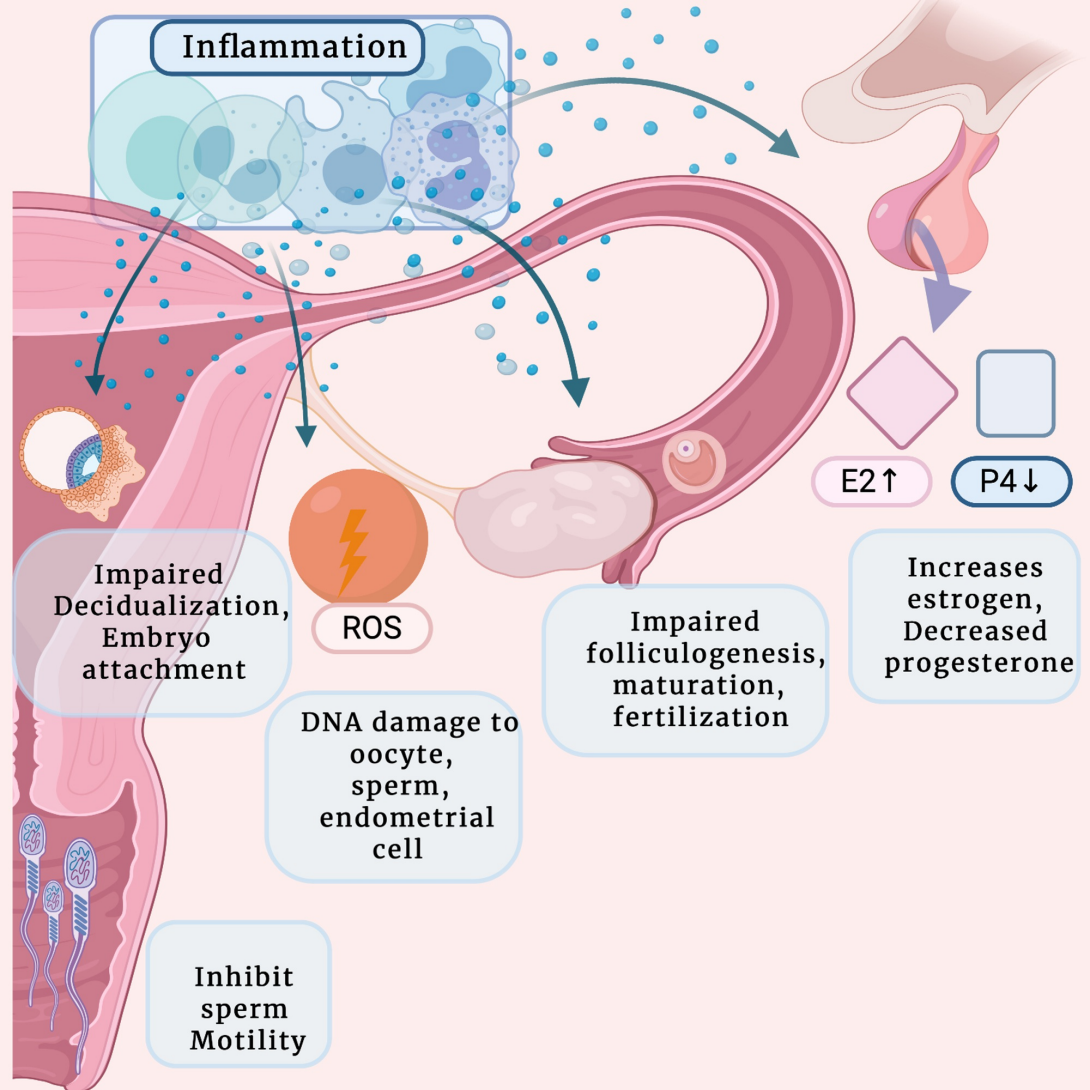
How inflammation causes infertility in endometriosis. E2: Estrogen; P4: progesterone; ROS: reactive oxygen species The inflammation will decrease infertility by: 1.Hormonal imbalance; 2.Impairing follicles maturation and fertilization; 3.Damaging the DNA, lipid, proteins of the oocyte, sperm, and other cells; 4.Inhibit implantation of the embryo; 5.Inhibiting sperm motility Created with BioRender.com

Inflammation distorts female reproductive function and conception through several aspects; these aspects are interconnected and complex, with some causing feedback loops on others (Figure 2).

First: Hormones

Ectopic endometrial lesions are believed to have increased sensitivity to estrogen since it stimulates the endometrium's proliferation, both inside and outside the uterine cavity. Thus promoting the development of endometriosis [36]. There is a balance between the inflammatory mediators and sex hormones required for the initiation and propagation of the physiological reproductive cycle, which includes folliculogenesis, ovulation, menstruation, embryo implantation, and pregnancy. Any disruption will negatively impact the reproductive function, one way of which is inflammation of endometriosis [29]. Hormonal alternation due to inflammation includes increased aromatase enzyme activity (which is responsible for producing estrogen) and changes in the sex steroid receptor expression. [34]

A review is done by (Miller et al., 2016) [29] to assess the immune dysfunction in endometriosis on infertility summed up the following findings:

Estrogen stimulates cyclooxygenase two enzyme (cox2) expression, which synthesizes prostaglandin E2 (PGE2). The production of this and cytokines shows to facilitate infertility in women with endometriosis.

Aberrant expressions of COX-2 and Aromatase P450 produce a local, continuous stream of estrogen and PGE2 in endometriosis patients [37], creating a state of estrogen dominance [38] and a positive feedback loop. Estrogen mediates the proliferation and differentiation of the granulosa cell and uterine lining; thus, it is responsible for the proliferative phase. As such, continual high expression of estrogen likely interferes with the transition from proliferative to secretory phase and other critical reproductive events. The state of estrogen dominance is also detrimental as estrogen is an inhibitor of αvβ3 integrin, a critical marker for endometrial receptivity in the uterine lining.

Not only estrogen but also progesterone receptors are affected in endometriosis. TNF-α and IL-1β are up-regulated in the peritoneal fluid (PF) of women with Endometriosis causing a decrease in the expression of progesterone receptor type B (PRB) mRNA in endometrial stromal cells isolated from women with Endometriosis [39]. This high expression of progesterone A receptor (PRA) relative to progesterone B receptor (PRB), has been found in the eutopic endometrium of women with endometriosis [29]. Reduced expression of PRB prevents appropriate progesterone signaling, and this progesterone resistance has been categorized as a "hallmark for implantation failure" since progesterone facilitates decidualization [38].

While treating endometriosis patients using hormonal intervention was previously emphasized, the regulation of inflammation and hormones seem interconnected and complicated as it is now well-established that the growth of endometriotic lesions is dependent on estrogen. It has been suggested that estrogens are delivered to the ectopic lesions in an endocrine fashion; however, new evidence suggests endometriotic lesions produce estrogen themselves. This feed-forward loop creates a myriad of cell signaling cascades in the peritoneal microenvironment that ends up compromising the physiological hormonal response [29].

Second: Oocyte Quality

Endometriosis has a negative effect on oocyte quality. The quality marker of the oocyte is reflected by the numbers of mature oocytes retrieved and the fertilization rates observed in the in vitro fertilization (IVF) outcomes [40-43]. Up to now, the literature proposes a reduced oocyte and embryo quality and an inferior pregnancy rate in women with endometriosis [29]. Inflammation is suggested to affect the oocyte quality indirectly by cytokines in peritoneal fluid, exerting an effect that modifies folliculogenesis and the oocyte spindles as well, which has consequences for both the maturation and fertilization of the oocyte [44]. Also, they have a lower cytoplasmic mitochondrial content compared to women who have infertility due to other causes [31].

In patients with moderate/severe endometriosis, the follicular environment was characterized by increased oxidative stress and leukocyte activation marker myeloperoxidase, and this increase is correlated with decreased oocyte quality and fertility [45]. Intra-follicular interleukin levels eight and twelve (IL-8, IL-12), and adrenomedullin are elevated in women with endometriosis undergoing IVF and are indicators of impaired embryo and oocyte quality [44]. PGE2 is involved in endometriosis's pathogenesis and affects oocyte maturation, ovulation, and fertilization [46].

Miller et al. reviewed articles to assess oocyte quality in endometriosis and found that while certain studies showed adverse effects, others have failed to find significant harm on oocyte quality between patients with endometriosis and controls; however, those lack the statistical power [29]. 

Third: Endometrial Receptivity

Endometrial receptivity is affected by endometriosis in different ways, but our focus here is how inflammation has a role in this. Lessey and Kim reviewed the major discoveries in endometriosis and related defects in endometrial receptivity and made a unifying concept regarding them [14]. The authors reviewed a large group of data suggesting that the endometrium of endometriosis patients is less receptive to implantation with a decrease cycle fecundity due to changes imposed by the disease. They stated that one of the contributors is low progesterone levels in endometriosis, mimicking the late phase of the menstrual cycle in which the progesterone level falls off, and a regulated inflammatory response begins thus in the setting of the disease this will create a premature inflammatory response. This blunted or inadequate response to progesterone in Endometriosis is due to low expression of the progesterone receptor and decrease expression in progesterone target genes. These blunted responses are created by the inflammatory process as explained earlier. 

Moreover, they suggest another means by which receptivity might be compromised, defective decidualization process due to a morphological change in the endometrium. The decidua plays a vital role in a successful maternal embryo interaction as it is required for providing nutrition, preventing immune rejection, and for trophoblast invasion. Thus, abnormal decidualization would have adverse effects on embryo implantation and pregnancy. It has also been mentioned that the systemic and local inflammatory cytokines disrupt the functionality of the endometrium and alteration in integrin and interleukins that are important for the attachment of the embryo [33].

Vannuccini et al. reviewed the effect of reproductive disorders on the pregnancy outcomes from a hormonal and inflammatory point of view, they suggested that the distorted cell proliferation and apoptosis, increased in oxidative stress, increased prostaglandin (PFE2 and PGF2ɑ), and the increase in cox2 expression will ultimately affect the decidualization reducing the endometrial receptivity and impacting the pregnancy outcome [30]. They also included the role of neuropeptides and neurohormones as they are part of the inflammatory setting, abnormalities in corticotropin-releasing hormone (CRH) and urocortin with impaired CRH receptor was shown in endometriosis women, hence a deranged decidualization process.

The endometrium of endometriosis patients with infertility is observed to have a high number of immature uterine natural killing cells (NK) compared to healthy fertile women. These subsets of NK cells live in the uterus and have a significant role in pregnancy and placenta formation [47].

Fourth: Sperm Function

A recent review done regarding the role of inflammation in endometriosis-associated infertility summarized what literature was found so far regarding inflammation and sperm motility [18]. It is the interleukins secreted from macrophages (IL6, IL8), macrophage migration inhibitory factor [MIF], and TNF alpha found in the peritoneal fluids that inhibit the sperm motility. Not only that, but they also affect the sperm DNA and the oocyte sperm binding and fusion (TNF alpha, MIF, IL1). Mast cells are hypothesized to cause a negative effect on the sperm motility by their mediators, one of which is the tryptase enzyme. However, another study done by Borelli et al. found that it is unlikely for tryptase to affect the sperm motility and that the strong interaction between the sperm and mast cell surface molecule causing significant degranulation might be related to endometriosis induced infertility by affecting the sperm in ways other than motility [48].

Fifth: Reactive Oxygen Species (ROS)

Many inflammatory diseases are associated with oxidative stress. Miller et al. reviewed the effect of the immune system and oxidative stress on inflammation [29]. The authors presented that oxidative stress offers a plausible mechanism to link inflammation and infertility. 

Máté G, Bernstein LR and Török AL also reviewed the role of oxidative stress in endometriosis. They suggested that macrophage and leukocyte invasion into the peritoneal fluid is a huge factor in the pathogenesis of Endometriosis induced infertility. These cells release free radicle through the respiratory burst NADPH oxidase system that directly causes damage to sperm, oocyte, and the embryo. IL6 and TNF alpha will induce the production of H2O2 via the same system. The refluxed blood also represents a source for a free radical generation. These free radicles are highly unstable; they cause damage to cellular organelles and molecular components (DNA, RNA, proteins, lipid, and carbohydrate), thus changing the cell structure and function and disrupting their miotic and mitotic divisions, which leads to cell death. They do so through lipid peroxidation, point mutation, and protein damage [32].

Máté G, Bernstein LR and Török AL also mentioned the role of antioxidants in this. Antioxidant systems can counteract these free radicles in the body. One of which is Glutathione. A high intracellular ratio of reduced glutathione to oxidized glutathione (GSH/GSSG ratio) is protective of microtubules [32]. Unfortunately, endometriosis patients exhibit abnormally low concentration of essential antioxidants, including glutathione, vitamin A, C, and E . Low levels of those antioxidants are associated with lower embryo quality and vice versa [49].

Both growth factors that are found abundantly in the peritoneal fluid and ROS induces fibrosis and adhesion, resulting in a loss of the ovarian stroma, thus affecting the blood supply and growth factors required for successful folliculogenesis [50]. Other causes of ROS are iron from the reflexed blood. 

We can see how these mechanisms overlap; ROS and hormonal imbalance can start the disease and generate inflammation resulting in damage to the oocyte, sperm, embryo, and receptivity. While inflammation itself will generate ROS and cause hormonal imbalance. This vicious cycle can be the core of infertility in endometriosis, especially in the early stages.

Inflammation and Therapeutic Targets for Endometriosis Induced Infertility

The available medical options for endometriosis only treat the symptoms rather than curing the disease. Medical treatment options include gonadotropin-releasing hormone (GnRH) agonists, oral contraceptives, aromatase inhibitors, and progestins. Lee D et al. reviewed the recent medical and surgical treatment of endometriosis-related infertility [17]. They suggested that hormonal therapy should be used as an adjunct to assisted reproductive technology since it has little effect or even it inhibits ovarian function according to previous studies. 

Lin et al. reviewed the current inflammatory therapeutic targets for infertility and classified them into 

A. Immunomodulators: include drugs that target the cytokines and ROS; however, most of these do not have enough data to be used for the management of Endometriosis [18].

B. Anti-cytokines: which also target the cytokines found in the peritoneal fluid-like (TNFalpha, IL6and TGF beta). Those cytokines can cause adhesion, damage the sperm, oocyte, and affect receptivity [18].

C. Statins: even though they are used in the treatment of hypercholesterolemia, have an anti-inflammatory effect by decreasing cell adhesion, chemokines thus inflammation [18].

D. Tyrosine kinase inhibitors: which inhibit the MAP kinase pathway and PI3/AKT that are involved in progesterone resistance and impaired decidualization [18].

E. Prostaglandin E2 inhibitors: we have discussed above how PGE2 can affect oocyte maturation, fertilization, and endometrial receptivity [18].

F. Antioxidants: it has been demonstrated how ROS can lead to reduced oocyte quality, affect sperm, and decrease receptivity [18].

Limitations

This study is a comprehensive review of other narrative reviews that provide information from the reviewer's angle. Quality assessment was not conducted. We did not include newer studies done using animal models. Older studies may have had more depth into the subject, but our focus was on new perspectives in the last five years. Our data was mainly collected from open access articles; valuable closed access articles may have been missed.

## Conclusions

Inflammation is an important cause of infertility in endometriosis. It impairs the decidualization, reduces the progesterone level, and disrupts the function of the endometrium. The hormonal imbalance created by the disease also plays a role as inflammation increases the aromatase activity, thus creating a dominant estrogen phase. The oocyte maturation and fertilization are affected through prostaglandins and cytokines from inflammatory cells. Not only the female reproductive system is affected but also the sperm entering through the uterus and fallopian tubes, have impaired motility, and their binding with the oocyte is impaired. Reactive oxygen species also cause damage to the endometrium, oocyte, and sperm by implying oxidative stress that causes an effect on a molecular level. These mechanisms provide insight into newer therapy techniques for infertility in endometriosis. Due to the burden of the disease, more clinical studies approaching anti-inflammatory strategies are needed to provide better evidence in the management of the disease.

## References

[1] Lee HJ, Park YM, Jee BC, Kim BY, Suh CS (2015). Various anatomic locations of surgically proven endometriosis: a single-center experience. Obstet Gynecol Sci.

[2] Alimi Y, Iwanaga J, Loukas M, Tubbs RS (2018). The clinical anatomy of endometriosis: a review. Cureus J Med Sci.

[3] Parasar P, Ozcan P, Terry KL (2017). Endometriosis: epidemiology, diagnosis and clinical management. Curr Obstet Gynecol Rep.

[4] Laganà AS, Garzon S, Götte M, Viganò P, Franchi M, Ghezzi F, Martin DC (2019). The pathogenesis of endometriosis: molecular and cell biology insights. Int J Mol Sci.

[5] Waldeyer Waldeyer (1870). The epithelial ovarian tumors, especially the cystomas (Article in German). Archives for gynecology.

[6] Gruenwald P (1942). Origin of endometriosis from the mesenchyme of the celomic walls. Am J Obstet Gynecol.

[7] Lauchlan SC (1972). The secondary müllerian system. Obstet Gynecol Surv.

[8] Dastur AE, Tank PD (2010). John A Sampson and the origins of endometriosis. J Obstet Gynaecol India.

[9] Brosens I, Benagiano G (2013). Is neonatal uterine bleeding involved in the pathogenesis of endometriosis as a source of stem cells?. Fertil Steril.

[10] Seli E, Berkkanoglu M, Arici A (2003). Pathogenesis of endometriosis. Obstet Gynecol Clin North Am.

[11] Deane JA, Gualano RC, Gargett CE (2013). Regenerating endometrium from stem/progenitor cells: is it abnormal in endometriosis, asherman’s syndrome and infertility?. Curr Opin Obstet Gynecol.

[12] Patel BG, Lenk EE, Lebovic DI (2018). Pathogenesis of endometriosis: interaction between endocrine and inflammatory pathways. Best Pract Res Clin Obstet Gynaecol.

[13] Prescott J, Farland LV, Tobias DK (2016). A prospective cohort study of endometriosis and subsequent risk of infertility. Hum Reprod Oxf Engl.

[14] Lessey BA, Kim JJ (2017). Endometrial receptivity in eutopic endometrium of women with endometriosis it is affected, let me show you why. Fertil Steril.

[15] Gao X, Yeh YC, Outley J, Simon J, Botteman M, Spalding Spalding (2006). Health-related quality of life burden of women with endometriosis: a literature review. Curr Med Res Opin.

[16] (2020). Fertility problems: assessment and treatment. https://www.nice.org.uk/guidance/cg156/chapter/Context.

[17] Lee D, Kim SK, Lee JR, Jee BC (2020). Management of endometriosis-related infertility: considerations and treatment options. Clin Exp Reprod Med.

[18] Lin YH, Chen YH, Chang HY, Au HK, Tzeng CR, Huang YH (2018). Chronic niche inflammation in endometriosis-associated infertility: current understanding and future therapeutic strategies. Int J Mol Sci.

[19] Wu M-H, Hsiao K-Y, Tsai S-J (2015). Endometriosis and possible inflammation markers. Gynecol Minim Invasive Ther.

[20] Reis FM, Petraglia F, Taylor RN (2013). Endometriosis: hormone regulation and clinical consequences of chemotaxis and apoptosis. Hum Reprod Update.

[21] Pellicer A, Albert C, Garrido N, Navarro J, Remohí J, Simón C (2000). The pathophysiology of endometriosis-associated infertility: follicular environment and embryo quality. J Reprod Fertil Suppl.

[22] Kang YJ, Jeung IC, Park A (2014). An increased level of IL-6 suppresses NK cell activity in peritoneal fluid of patients with endometriosis via regulation of SHP-2 expression. Hum Reprod Oxf Engl.

[23] Zondervan KT, Becker CM, Missmer SA (2020). Endometriosis. N Engl J Med.

[24] Symons LK, Miller JE, Kay VR (2018). The Immunopathophysiology of endometriosis. Trends Mol Med.

[25] Ziegler D de, Borghese B, Chapron C (2010). Endometriosis and infertility: pathophysiology and management. The Lancet.

[26] Wang XM, Ma ZY, Song N (2018). Inflammatory cytokines IL-6, IL-10, IL-13, TNF-α and peritoneal fluid flora were associated with infertility in patients with endometriosis. Eur Rev Med Pharmacol Sci.

[27] Gica N, Panaitescu AM, Iancu G (2020). The role of biological markers in predicting infertility associated with non-obstructive endometriosis. Ginekologia Polska.

[28] Armour M, Lawson K, Wood A, Smith CA, Abbott J (2019). The cost of illness and economic burden of endometriosis and chronic pelvic pain in Australia: a national online survey. PLOS ONE.

[29] Miller JE, Ahn S, Monsanto SP, Khalaj K, Koti M, Tayade C (2016). Implications of immune dysfunction on endometriosis associated infertility. Oncotarget.

[30] Vannuccini S, Clifton VL, Fraser IS, Taylor HS, Critchley H, Giudice LC, Petraglia F (2016). Infertility and reproductive disorders: impact of hormonal and inflammatory mechanisms on pregnancy outcome. Hum Reprod Update.

[31] Sanchez AM, Vanni VS, Bartiromo L (2017). Is the oocyte quality affected by endometriosis? A review of the literature. J Ovarian Res.

[32] Máté G, Bernstein LR, Török AL (2018). Endometriosis is a cause of infertility. Does reactive oxygen damage to gametes and embryos play a key role in the pathogenesis of infertility caused by endometriosis?. Front Endocrinol.

[33] Yu J, Berga SL, Zou W, Taylor RN (2019). Interleukin-1β inhibits estrogen receptor-α, progesterone receptors A and B and biomarkers of human endometrial stromal cell differentiation: implications for endometriosis. Mol Hum Reprod.

[34] García-Gómez E, Vázquez-Martínez ER, Reyes-Mayoral C, Cruz-Orozco OP, Camacho-Arroyo I, Cerbón M (2020). Regulation of inflammation pathways and inflammasome by sex steroid hormones in endometriosis. Front Endocrinol.

[35] Hill CJ, Fakhreldin M, Maclean A (2020). Endometriosis and the fallopian tubes: theories of origin and clinical implications. J Clin Med.

[36] Augoulea A, Alexandrou A, Creatsa M, Vrachnis N, Lambrinoudaki I (2012). Pathogenesis of endometriosis: the role of genetics, inflammation and oxidative stress. Arch Gynecol Obstet.

[37] Santulli P, Marcellin L, Tosti C (2015). MAP kinases and the inflammatory signaling cascade as targets for the treatment of endometriosis?. Expert Opin Ther Targets.

[38] Fox C, Morin S, Jeong JW, Scott RT Jr, Lessey BA (2016). Local and systemic factors and implantation: what is the evidence?. Fertility and Sterility.

[39] Grandi G, Mueller MD, Papadia A (2016). Inflammation influences steroid hormone receptors targeted by progestins in endometrial stromal cells from women with endometriosis. J Reprod Immunol.

[40] Giacomini E, Sanchez AM, Sarais V, Beitawi SA, Candiani M, Viganò P (2017). Characteristics of follicular fluid in ovaries with endometriomas. Eur J Obstet Gynecol Reprod Biol.

[41] Hamdan M, Dunselman G, Li TC, Cheong Y (2015). The impact of endometrioma on IVF/ICSI outcomes: a systematic review and meta-analysis. Hum Reprod Update.

[42] Rossi AC, Prefumo F (2016). The effects of surgery for endometriosis on pregnancy outcomes following in vitro fertilization and embryo transfer: a systematic review and meta-analysis. Arch Gynecol Obstet.

[43] Shebl O, Sifferlinger I, Habelsberger A, Oppelt P, Mayer RB, Petek E, Thomas E (2017). Oocyte competence in in vitro fertilization and intracytoplasmic sperm injection patients suffering from endometriosis and its possible association with subsequent treatment outcome: a matched case-control study. Acta Obstet Gynecol Scand.

[44] Singh AK, Dutta M, Chattopadhyay R, Chakravarty B, Chaudhury K (2016). Intrafollicular interleukin-8, interleukin-12, and adrenomedullin are the promising prognostic markers of oocyte and embryo quality in women with endometriosis. J Assist Reprod Genet.

[45] Santanam N, Zoneraich N, Parthasarathy S (2017). Myeloperoxidase as a potential target in women with endometriosis undergoing ivf. Reprod Sci Thousand Oaks Calif.

[46] Sacco K, Portelli M, Pollacco J (2012 Feb 1). The role of prostaglandin E2 in endometriosis. Gynecol Endocrinol.

[47] Thiruchelvam U, Wingfield M, O’Farrelly C (1989). Increased unk progenitor cells in women with endometriosis and infertility are associated with low levels of endometrial stem cell factor. Am J Reprod Immunol N Y N.

[48] Borelli V, Martinelli M, Luppi S (2020). Mast cells in peritoneal fluid from women with endometriosis and their possible role in modulating sperm function. Front Physiol.

[49] Choi YS, Cho S, Seo SK, Park JH, Kim SH, Lee BS (2015). Alteration in the intrafollicular thiol-redox system in infertile women with endometriosis. Reprod Camb Engl.

[50] Hsueh AJW, Kawamura K, Cheng Y, Fauser BCJM (2015). Intraovarian control of early folliculogenesis. Endocr Rev.

